# Interleukin-6 Signaling Pathway and Its Role in Kidney Disease: An Update

**DOI:** 10.3389/fimmu.2017.00405

**Published:** 2017-04-21

**Authors:** Hua Su, Chun-Tao Lei, Chun Zhang

**Affiliations:** ^1^Department of Nephrology, Union Hospital, Tongji Medical College, Huazhong University of Science and Technology, Wuhan, China

**Keywords:** IL-6 classic signaling, IL-6 trans-signaling, podocyte, mesangial cells, endothelial cells, tubular epithelial cells, renal disease

## Abstract

Interleukin-6 (IL-6) is a pleiotropic cytokine that not only regulates the immune and inflammatory response but also affects hematopoiesis, metabolism, and organ development. IL-6 can simultaneously elicit distinct or even contradictory physiopathological processes, which is likely discriminated by the cascades of signaling pathway, termed classic and trans-signaling. Besides playing several important physiological roles, dysregulated IL-6 has been demonstrated to underlie a number of autoimmune and inflammatory diseases, metabolic abnormalities, and malignancies. This review provides an overview of basic concept of IL-6 signaling pathway as well as the interplay between IL-6 and renal-resident cells, including podocytes, mesangial cells, endothelial cells, and tubular epithelial cells. Additionally, we summarize the roles of IL-6 in several renal diseases, such as IgA nephropathy, lupus nephritis, diabetic nephropathy, acute kidney injury, and chronic kidney disease.

## Introduction

Interleukin-6 (IL-6) was discovered in 1986 as a B cell stimulatory factor initiating IgG production (1). Later, it was demonstrated to be a multifunctional cytokine that regulates numerous biological processes including the organ development, acute-phase responses, inflammation, and immune responses (2).

Up to date, ten IL-6 family cytokines have been identified: IL-6, oncostatin M (OSM), leukemia inhibitory factor (LIF), ciliary neurotrophic factor (CNTF), cardiotropin-1 (CT-1), cardiotrophin-like cytokine (CLC), neuropoietin (NP), IL-11, IL-27, and IL-31 (3–5). All these cytokines share the membrane glycoprotein gp130 as a common receptor and signal transducer subunit in their receptor complexes except IL-31. The formation of the signal-transducing complex of each cytokine depends on specific ligand and receptor association. IL-6 and IL-11 bind to the specific membrane-bound α chain of IL-6 receptor (IL-6R) or IL-11R, respectively, and then connect to gp130, leading to the homodimerization of gp130. CLC, CT-1, OSM interact with gp130/LIFR or gp130/OSMR and then form heterodimeric receptor complexes to conduct signal. For IL-31, it utilizes the IL-31R and OSMR as signal-transducing receptor, and it is the only cytokine of IL-6 family that does not need gp130 for signal transmission [Figure S1 in Supplementary Material quoted from Garbers et al. (6)].

The IL-6 family cytokines show diverse physiological and pathological functions. Some of their functions are overlapping such as both IL-6 and IL-11 participate in promoting the synthesis of acute-phase protein in hepatocytes (7–9), and IL-11, IL-6, OSM as well as CT-1 are all involved in the bone metabolism by stimulating osteoclast formation (10, 11). However, more frequently, individual IL-6 family members have distinct functions by the respective specific receptors that may not present simultaneously or spatially restricted. IL-6 is the one that has been studied most, and we will mainly focused on it in this review.

## Classic and Trans-Signaling Pathway of IL-6

On target cells, IL-6 first binds to the α-chain of non-signaling membrane-bound IL-6R (mbIL-6R, also named CD126 or gp80); subsequently, this complex connects to two molecules of gp130 and initiates signal transduction. JAK/STAT3 and SHP2/Gab/MAPK are the two major pathways involved in gp130 signaling which is activated *via* the YxxQ motif and Y759 of gp130, respectively (5, 12–14).

It is worthwhile to note that only a few cell types, such as macrophages, neutrophils, CD4^+^ T-cells, podocytes, and hepatocytes, express IL-6R on the cell surface, and therefore can directly respond to IL-6 (15, 16).

Soluble receptors have been identified for many cytokines and are pivotal regulators by acting as agonists or antagonists of cytokine signaling and inflammatory events. For example, the soluble receptors of TNF-α and IL-1α act as antagonists by neutralizing and inhibiting the activity of their ligands (17–19). A soluble form of the IL-6R (sIL-6R) has been detected in body fluids such as blood and urine (20). sIL-6R binds to IL-6 with comparable affinity as the mbIL-6R (21); consequently, the complex of IL-6/sIL-6R can activate gp130, a membrane protein is ubiquitously expressed (22–25). Activation of gp130 *via* the IL-6/sIL-6R complex is termed IL-6 trans-signaling pathway, whereas activation of gp130 *via* the mbIL-6R is named IL-6 classic signaling pathway (23, 26–28). IL-6 trans-signaling represents an alternative to classic IL-6 signaling and permits IL-6 to modulate a broad spectrum of target cells [Figure S2 in Supplementary Material, quoted from Ref. (29)].

Due to the ubiquitous expression of gp130 and the extensive involvement of IL-6 trans-signaling pathway in diverse physiologic and pathologic processes, to get a comprehensive understanding of the generation of sIL-6R is critical. In humans, the sIL-6R is generated *via* two distinct mechanisms. The first one implicates proteolytic cleavage of the mbIL-6R and relies on a metalloprotease activity, and the second mechanism is by the translation of a differentially spliced IL-6R mRNA lacking the transmembrane and cytosolic domains (30–32). It is believed that the generation of the sIL-6R is mainly regulated by metalloprotease cleavage rather than *via* differential mRNA splicing (33). In the mouse, only shedding *via* enzymatic digestion but no differential splicing of the IL-6R mRNA was observed (30). The shedding of the IL-6R is catalyzed by Zn2^+^-metalloproteases of the ADAM (a disintegrin and metalloprotease) family (34, 35), among which ADAM10 and ADAM17 are the most related enzymes that driving IL-6R proteolysis. It is documented that ADAM10 mediates the slow constitutive IL-6R cleavage, while ADAM17 is account for rapid IL-6R shedding upon diverse stimulations (36). Many factors can activate ADAM17, such as pro-inflammatory cytokines (IL-1β and TNF-α), bacterial toxins, cellular cholesterol depletion, PKC agonist, proteasome inhibitor, DNA damage, and so on (34, 36–40). Therefore, modulation of ADAM17 activity is essential for the IL-6 trans-signaling conduction.

Soluble form of gp130 (sgp130) is found naturally produced and it is detected in the circulation at relatively high concentration (100–400 ng/ml in human plasma) (4, 41–44). Unlike sIL-6R, sgp130 is predominantly generated by alternative splicing rather than proteolysis (45). Since sgp130 can interact with the IL-6/sIL-6R complex, it acts as a specific inhibitor of IL-6 trans-signaling pathway (46, 47), whereas it does not affect mbIL-6R-mediated classic signaling. Notably, sgp130 is specific for the IL-6/sIL-6R complex due to signaling of other IL-6 family cytokines like LIF and OSM were blocked at 100–1,000-folds higher concentrations and CNTF and IL-27 signaling were not affected at all (46, 48, 49). Experimentally, sgp130 can be utilized as molecular tool to discriminate between IL-6 classic signaling and trans-signaling because it competitively inhibits trans-signaling without affecting classic signaling.

Global IL-6 signaling can be halted by IL-6 or IL-6R neutralizing antibodies. In parallel experiments, IL-6 trans-signaling can be inhibited with the sgp130Fc protein, which could be provided by either injection of the recombinant protein or generation by sgp130Fc transgenic mice. Moreover, IL-6 trans-signaling can be also activated with hyper-IL-6 (recombinant soluble IL-6/sIL-6R fusion protein). The experimental design using these approaches could elucidate whether the IL-6-gp130-initiated effect is mediated by IL-6 classic or trans-signaling pathway (23, 50).

The property of IL-6 during inflammation process is complicated, in addition to its well-known pro-inflammatory properties, it also elicits anti-inflammatory effects under certain situations. Usually, it is believed that IL-6 classic signaling is anti-inflammatory whereas the trans-signaling is pro-inflammatory (51). In detail, IL-6 classic signaling is implicated in the synthesis of acute-phase proteins in hepatocyte, which have anti-inflammatory properties and are indispensable for immune defense (52). Whereas, in various inflammation and autoimmune diseases, as well as inflammation-associated cancer, IL-6 trans-signaling is pro-inflammatory and blockade of it is sufficient to alleviate the inflammatory reaction (53–61). But the controversy is existed. Several investigations showed that abrogating IL-6 trans-signaling cannot provide protective effects (28), even unexpectedly attenuated the recovery processes of the disease under certain conditions. For example, IL-6 trans-signaling is critical to regulate the transition from neutrophil to monocyte and prevent excessive tissue damage (62). Therefore, it appears in different diseases that the pathogenetic roles of IL-6 classic and trans-signaling are distinct.

## IL-6 and Immune and Inflammatory Cells

### T Lymphocytes

Growing data conclusively confirmed that IL-6 is involved in the regulation of T cell differentiation between two critical CD4^+^ T cell populations: regulatory T (Treg) cells and T helper 17 (Th17) cells. Specifically, IL-6 triggers the differentiation of Th17 cells together with TGF-β by enhancing RORγt expression (63–66), while it dampens the generation of Treg cells *via* STAT3 (67–70). Th17 cells secrete a number of pro-inflammatory cytokines, such as IL-17, and initiate various inflammatory responses. Meanwhile, diminished Treg further worsen the situation.

Besides that, it is well documented that IL-6 signaling promotes T cells proliferation and resists its apoptosis by producing IL-2 and activating STAT3 (53, 71, 72), and it also activates Th2 cytokine generation through the transcription factor C/EBP (73). Furthermore, follicular helper T cells (T_FH_), a newly identified CD4^+^ T helper subpopulation, which is differentiated from naive CD4^+^ T cells in the presence of IL-6, and is a strong inducer for B cell activation (74).

Therefore, IL-6 is clearly implicated in CD4^+^ T cells differentiation and expansion and plays a key role in the T-cell-mediated immune response. Additionally, IL-6 is indirectly involved in B cell-induced inflammation *via* T_FH_. Targeting IL-6 signaling is promising for the treatment of autoimmune and inflammatory Diseases.

### Monocyte and Macrophage

It is well known that IL-6 modulates monocytes differentiation between macrophages and dendritic cells (DCs). Macrophages play an essential role in inflammatory response by secreting cytokines, chemokines, and matrix metalloproteinases. Local macrophages are differentiated from peripheral blood, with the exposure of GM-CSF, and IL-4 peripheral blood monocytes differentiate into DCs, whereas the additional IL-6 switches the differentiation of monocytes from DCs to macrophages. This switch is caused by IL-6-mediated upregulation of M-CSF receptors on monocytes (75, 76). However, this effect is not seen with the addition of other IL-6 family members, including IL-11, LIF, and OSM (77).

Interleukin-6 can trigger myeloid leukemia cell to express complement receptors and Fc receptors along with F4/80 (a marker for mature macrophages) (78–80). In addition, IL-6 stimulates various macrophages typical gene expression, including early response genes c-Jun, Jun B, Jun D, JAK3, Egr-1, and late response genes, such as lysozyme and ferritin light-chain (78, 80). Consistently, upon IL-6 stimulation, monocytic cells upregulate MCP-1 mRNA and protein, a chemokine more strongly expressed in macrophages rather than monocytes (81).

Moreover, gp130 and downstream STAT3 activation are required for IL-6-induced macrophage differentiation, because gp130 mutation and dominant-negative form of STAT3 can block it (82, 83). Thus, IL-6-mediated gp130-JAK/STAT3 signaling drives macrophage differentiation from monocyte.

In contrast to abundant evidence *in vitro*, there is little data confirmed that IL-6 plays a role in macrophage differentiation *in vivo*. Macrophages from IL-6-deficient and wild-type mice are similar, except for the decreased capability to defend microorganism (84–86). Hence, other factors compensating for IL-6 may induce macrophage differentiation. So IL-6 appears to be sufficient, but not necessary for macrophage differentiation *in vivo*.

### Local Generation and Signaling of IL-6 in Kidney

Emerging data showed that local activation of IL-6 classic and trans-signaling pathway is implicated in renal autoimmune and inflammatory diseases. Kidney resident cells, including podocytes, endothelial cells, mesangial cells, and tubular epithelial cells (TECs) can secrete IL-6 under certain milieu. Podocyte is the only resident cell that expresses IL-6R, while others do not express IL-6R and not employ classic IL-6 signaling (87, 88) (Table 1).

**Table 1:** **Effect of IL-6 on kidney cells**.

Type of cells	Production of IL-6	Expression of mbIL-6R	Expression of gp130	Effect of IL-6
Podocyte	Yes	Yes	Yes	Promotes proliferation; affects the differentiation, cell cycle^a^
Mesangial cell	Yes	No	Yes	Enhances proliferation, matrix accumulation; increases MCP-1 synthesis and release^b^
Endothelial cell	Yes	No	Yes	Induces vasoconstriction and endothelial dysfunction; increases ROS production^c^
Tubular epithelial cell	Yes	No or weak	Yes	Stimulates tubular atrophy; increases collagen I generation; accelerates tubulointerstitial fibrosis^d^

*^a^Moutabarrik et al. (15); Feng et al. (89); Lu et al. (90); Dai et al. (91)*.

*^b^Coletta et al. (87); Gohda et al. (92); Lu and Zhou (93)*.

*^c^Wassmann et al. (94); Schrader et al. (95)*.

*^d^Ranganathan et al. (96); Kielar et al. (97); Harcourt et al. (98)*.

### IL-6 and Podocytes

Podocyte is a well-known source of IL-6, under serum-deprived condition, there is no detectable IL-6 in the supernate of cultured podocytes, but with the exposure to pro-inflammatory mediators, such as LPS, TNF-α, and IL-1β, IL-6 was detected and its concentration was increased in a dose- and time-dependent manner (99–102). It was reported that dexamethasone and vitamin D can suppress IL-6 expression, which partially accounts for their anti-inflammation effects (103–105). Interestingly, podocyte is one of the limited number of cells that can express IL-6R and its abundance was also upregulated by pro-inflammatory stimulation. However, the soluble form of IL-6R (sIL-6R) was not identified in the culture supernatants collected from unstimulated or cytokine-stimulated cells. As we mentioned before, ADAM10 and ADAM17 are the key enzymes cleaving mbIL-6R and generating sIL-6R, thus maybe upregulation of ADAM activity could induce sIL-6R production; however, it requires further investigation. In addition, IL-6 promotes podocyte proliferation in an autocrine fashion (15).

Besides pro-inflammatory factors, high glucose also increased IL-6 secretion and triggered IL-6 signal transduction in podocytes which was abrogated by IL-6-neutralizing antibody or IL-6 siRNA (100). The author suggested that blocking IL-6 and its downstream mediators such as IL-6R and gp130 may attenuate the progression of diabetic nephropathy (DN).

More recently, Abkhezr (106) observed that upon 100 nM angiotensin II (Ang II) exposure for 6–24 h, the STAT3 was phosphorylated *via* gp130-mediated signaling in podocytes; however, it seems not by classic IL-6 pathway. Intriguingly, these effects were abrogated by TRPC6 gene depletion as well as by inhibitors of the Ca^2+^-dependent downstream enzymes calcineurin and Ca^2+^-calmodulin-dependent protein kinase II (CaMKII). It is well known that the STAT3 activity is positively correlated with glomerular cell proliferation in glomerular disease (107). Conditional deletion of STAT3 in podocyte ameliorated the glomerular and tubulointerstitial insults in several animal models (89–91). Taken together, it is suggested that IL-6 trans-signaling initiated by Ang II could activate STAT3 in podocytes in a TRPC6-dependent manner which consequently affect the differentiation, cell cycle, and other physiopathologic processes of podocytes.

Notably, apart from above detrimental role in glomerular diseases, soluble IL-6 generated by podocytes also has anti-inflammation function which was demonstrated in co-culture system of podocytes and endothelial cells. Specifically, TNF-α stimulated podocyte to secrete IL-6 which upregulated the expression of suppressor of cytokine signaling 3 in glomerular endothelium and elicited the immunosuppressive action of IL-6, consequently reduced the recruitment of neutrophils to endothelium (108); however, this issue remains debatable.

Collectively, podocyte is a vigorous origin of IL-6, the local excessive expression of IL-6, and its receptor may actively involve in the process of diverse glomerular diseases in an autocrine and/or paracrine fashion. However, its detailed roles in the development and resolution of the insults require further investigation. The discrepancy between reports may explain by the different experiment setting and different downstream signal pathway involved. Debate about IL-6 function also exists in other inflammatory diseases, as data propose that it serves as a marker rather than a mediator of inflammation (109).

### IL-6 and Mesangial Cells

Under certain situation, mesangial cells also can secrete IL-6 and activate inflammatory cells which play an essential role in immune and metabolism-mediated injury of kidney (110–112). As we know, mesangial cells only express the gp130 and not the IL-6R. Exposure to IL-6 and sIL-6R together strongly promoted mesangial cells to synthesize and release monocyte chemoattractant protein 1 and subsequently enhance monocyte recruitment (87). Whereas IL-6 or sIL-6R alone was ineffective to induce cytokine or chemokine secrete from mesangial cells.

Additionally, IL-6 involves in pathological abnormalities of mesangium by enhancing its proliferation, matrix accumulation, and sclerosis (92, 93).

### IL-6 and Endothelial Cells

Inflammatory stimuli, specifically, IL-1, LPS, TNFα, and IL-4 are the common inducers for endothelial cells to generate IL-6. IL-6 promotes Ang II type 1 receptor (ATR1) gene expression and leads to Ang II-induced vasoconstriction and ROS production which ultimately results in endothelial dysfunction (94). Consistently, IL-6 deficiency mice are protected against Ang II-mediated endothelial dysfunction (95).

Endothelial cell does not express transmembrane IL-6R, thus IL-6 merely *via* trans-signaling pathway regulates endothelial function (113). Interplay between IL-6 and endothelial cells modulates leukocytes recruitment and chemokine secretion. IL-6 knockout mice showed reduced leukocyte aggregation at inflammatory sites, which is associated with diminished endothelial surface adhesion molecules expression and chemokines production (114).

### IL-6 and Tubular Epithelial Cells

Numerous systemic or local insults, including hypoxemia, nephrotoxin, oxidized lipid, advanced glycation end products, immune complexes, cytokines, and chemokines, could initiate TEC to synthesize and secrete IL-6. For example, anti-dsDNA antibodies can induce IL-6 *de novo* synthesis in proximal TEC. Interesting, for IL-6 secretion, TEC is more sensitive to anti-dsDNA antibodies compared to mesangial cells (115). Notably, glomerular injury is a potent inducer for IL-6 generation in TEC which presents one aspect of glomeruli-tubules cross talk.

It is shown that IL-6 can trigger proximal TEC to generate the collagen I and accelerate tubulointerstitial fibrosis, which was associated with enhanced STAT3 phosphorylation. Suppression of IL-6 expression in the TEC hampered the interstitial fibrosis and tubular atrophy whereas chronic administration of IL-6 enhanced the fibrotic process (96). Although many studies favor that IL-6 contributes to acute and chronic kidney injury and fibrosis, opposing opinions still exist (97, 98).

### IL-6 and Renal Disease

#### IL-6 and IgA Nephropathy (IgAN)

Local deposited high-molecular polymeric IgA1 can promote mesangial cells proliferation and secretion of the pro-inflammatory cytokine IL-6 (111, 116, 117). Moreover, MAPK/ERK signaling, apparently relying on AT1R activation, is implicated in mesangial cells IL-6 secretion. Because losartan, an AT1R blocker can block MAPK/ERK signaling and IL-6 production from mesangial cells. Thus, utilizing RAS blockers to treat IgAN is rationale and promising (118). Upon complement system is activated by immune complex, sublytic C5b-9 can stimulate IL-6 and TGF-β1 secretion in mesangial cells in a p300-C/EBPβ-dependent manner which was demonstrated in mesangioproliferative glomerulonephritis (MPG) model. In parallel, the Th17-associated cytokines in serum and urine were elevated and correlated with renal pathological change. Additionally, locally, blockage of IL-6 generation by p300 and C/EBPβ gene silence protected MPG model from renal injury, including the reduced Th17 cytokines, ameliorated mesangial cells proliferation, ECM accumulation, and diminished proteinuria (119). Therefore, in IgAN, immune complex and complement component both can stimulate mesangial cell to secrete IL-6 and ensuing itself proliferation and inflammatory cell recruitment.

#### IL-6 and Lupus Nephritis (LN)

In systemic lupus erythematosus (SLE) patients and mice, the concentration of IL-6 is elevated in serum, urine, and glomeruli, which positively associated with disease activity (120–126). B lymphocytes of SLE subjects spontaneously produced anti-dsDNA autoantibody which was halted by IL-6 inhibition meanwhile it was regained with exogenous supply of IL-6 (120). As we know, CD5 is a suppressor of B cell receptor signaling and negatively regulated B cells activity, meantime IL-6 can dampen CD5 expression *via* DNA methylation and consequently initiate the activation and expansion of autoreactive B cell lineage (127).

Interleukin-6 gene polymorphism (IL-6 174G>C) on the promoter region was found to convey the susceptibility of SLE in certain ethnical groups, such as in Caucasians. However, it is not identified in Asian population (128–130).

Besides its systemic effects, IL-6 was proved to have a strong correlation with the activity of LN. It was shown that elevated urinary IL-6 excretion was associated with a higher activity of LN (131, 132). In addition, local IL-6 expression within the glomeruli and tubules was evidently increased in LN (133). In SLE animal model, IL-6-deficient MRL-*Fas^lpr^* mice were resistant to immune- and inflammatory-mediated tissue injury accompanying with delayed onset of proteinuria and hematuria. The lack of IL-6 led to marked reduction of macrophages, CD4^+^ and CD8^+^T lymphocytes infiltrates in the kidney, a reduction of IgG deposition, and C3 fixation in kidney (122). In another lupus-prone mice, Lyn^−/−^ mice, inhibition of IL-6 trans-signaling by sgp130Fc exerted relatively little effect on abnormal immune processes along with unchanged pathogenic autoantibodies and renal immune complexes deposition, whereas sgp130Fc indeed ameliorated glomerulonephritis and preserved renal function by hampering complement fixation, leukocytes infiltration, and macrophage expansion in this model (134).

Collectively, IL-6 is a vigorous player in LN and will be a promising therapeutic target.

#### IL-6 and DN

High IL-6 level was identified in early stage of type 1 diabetes proposing IL-6 is involved in islet cells impairment. Additionally, an IL-6-inducible autoimmunity-related gene (HIP/PAP) was found to be expressed in the pancreas in patients with type-1 diabetes further indicating a potential correlation between IL-6 and autoimmune diabetes (135). In type 2 diabetes, IL-6 level was increased and associated with atherosclerosis development (136). *In vitro* IL-6 induced insulin resistance which support its role in type 2 diabetes occurrence (137, 138).

Chronic inflammatory process participates in the development of microvascular complications of diabetes. DN patients showed an elevated serum level of inflammatory cytokines, including IL-6, which positively correlated with the extent of proteinuria (139, 140). Meantime, hyperglycemia can trigger podocytes, mesangial cells, interstitial tissue, and tubules to generate IL-6 which contribute to local and systemic inflammatory process in DN (110, 141).

Apart from its involvement in LN, IL-6 gene polymorphism also conveys the susceptibility to cancer (142), lipid metabolic abnormalities, and inflammatory disorders (143). Recently, it was found that IL-6 gene 174G>C polymorphism is an independent risk factor for DN in Turkish and Greek type 2 diabetic mellitus patients (144, 145). Therefore, it again suggested that IL-6 is a cardinal player in DN.

#### IL-6 and Acute Kidney Injury (AKI)

Recent studies have shown a close correlation between IL-6 expression and AKI. In ischemic AKI animal model, it was found that IL-6 transcription and signaling are elevated locally and systemically after 60 min bilateral kidney ischemia. This finding indicated that IL-6 signaling connected local and systemic inflammation and can be employed as a biomarker and therapeutic target in ischemic AKI (146).

Similarly, in nephrotoxin-induced AKI, IL-6 expression was dramatically enhanced in kidney (113-fold), predominantly in renal TECs, and strongly correlated with the damage of kidney. While IL-6 deficiency attenuated neutrophil accumulation and caused mice relatively resistant to the insult. Moreover, neutrophil depletion in wild-type mice markedly reduced nephrotoxin-induced injury as well. Thus, IL-6-mediated neutrophil activation is one of the central mechanisms for AKI. Intriguingly, stimulation of IL-6 trans-signaling significantly mitigated renal damage and preserved renal function *via* underlying anti-oxidative stress mechanism (88). The similar observation was reported in ischemia-reperfusion-induced AKI model which proposed that IL-6 trans-signaling may play a protective role by promoting repair process (147, 148).

#### IL-6 and Chronic Kidney Disease (CKD)

The elevated plasma IL-6 level is commonly observed in CKD patients (149), which is largely caused by the increased generation resulting from oxidative stress, chronic inflammation, and fluid overload. Meanwhile, the reduced clearance of IL-6 due to the impaired renal function also contributes to its accumulation. In the end stage renal disease (ESRD) patients, the therapeutic hemodialysis and peritoneal dialysis *per se* further stimulate inflammatory responses and increase IL-6 production (150, 151).

Interleukin-6 accelerates the progression of CKD not only by aggravating kidney injury as described above but also by initiating its complications, especially the chronic vascular disease (CVD). It is demonstrated that IL-6 initiates the endothelial injury mainly *via* reducing endothelial nitric oxide synthase (eNOS) and adiponectin (an anti-atherogenic adipokine) expression (152), and the injection of recombinant IL-6 exacerbates atherosclerosis (153); these findings suggest that IL-6 also contributes to the increased incidence of CVD in CKD patients.

Taken together, elevated IL-6 level is not only a consequence of CKD, more importantly, it also acts as a trigger for the progression of CKD and its related complications.

## Conclusion and Perspective

In brief, IL-6 could be produced by renal resident cells, including podocytes, mesangial cells, endothelial cells, and TECs. Meantime, all these cells, as well as immune and inflammatory cells will actively respond to IL-6 *via* classic or/and trans-signaling pathway. It has already been evidently elucidated that IL-6 participates in renal instinct cell injury and repair process, as well as a variety of immune, metabolic, ischemic, and toxemic-mediated renal diseases.

More importantly, IL-6R-neutralizing mAb tocilizumab (Actemra) has already been approved for treatment in patients with certain autoimmune diseases, such as rheumatoid arthritis in more than 100 countries, including European Union, United State, Brazil, and India. And in Japan, it was authorized to be used in patients with juvenile idiopathic arthritis and Castleman’s disease (154). Several second generation of IL-6 inhibitors, including anti-IL-6 antibodies, is under development.

Intriguingly, recently it was found that cardiotrophin-like cytokine-1 (CLC-1), a member of the IL-6 family and transmitting signal *via* gp130, is almost probable permeability factor of FSGS. Strikingly, the concentration of CLC-1 is up to 100 times higher in the circulation of FSGS patients compared to normal subjects (155). *In vitro* CLC-1 can mimic the effects of plasma from FSGS patients on albumin permeability, and it reduces nephrin expression in glomeruli and cultured podocytes which can be prevented by CLC-1 monoclonal antibody.

Thus, further investigation of IL-6 and its family members as well as its signaling pathway are imperative for getting a full view of kidney disease and developing more effective drugs.

## Author Contributions

Dr. CZ organized and approved the final version of this review; Dr. HS organized and drafted this review; Dr. C-TL drafted this review.

## Conflict of Interest Statement

The authors declare that the research was conducted in the absence of any commercial or financial relationships that could be construed as a potential conflict of interest.
