# Supplementary material for: Interleukin-6 Signaling Pathway and Its Role in Kidney Disease: An Update
Source: Front Immunol. 2017 Apr 21;8:405. doi: 10.3389/fimmu.2017.00405 (PMC5399081; doi:10.3389/fimmu.2017.00405)
Supplement: Supplementary file 1 [file Image_1.PDF]

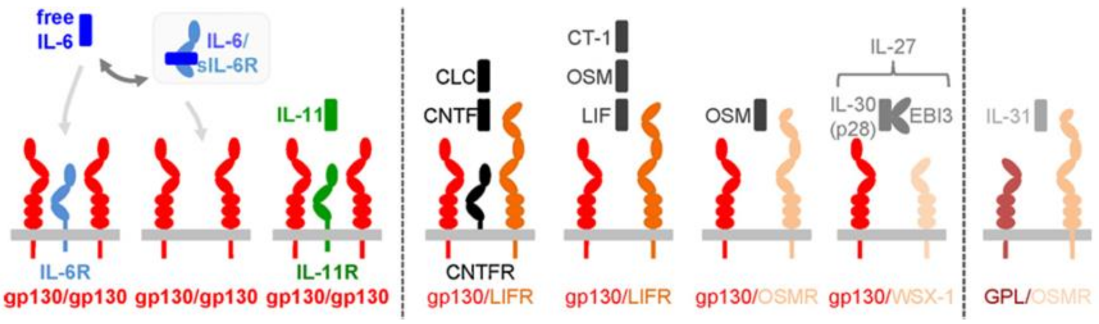

Supplementary figure 1. Receptor complexes of the IL-6-type family of cytokines. All cytokines of the gp130-signaling family use gp130 (red) as at least one part of the signal-transducing β-receptor complex. Some cytokines need additional β-receptor molecules directly involved in signal transduction into the cell LIF-R (dark orange), OSM-R (orange), WSX-1 (wheat), GPL (dark red) or non-signaling α-receptors (IL-6R (blue), IL-11-R (green), CNTF-R (dark grey), EBI3 (grey). Cytokines of this family are IL-6 (dark blue), IL-11 (green), CNTF (black), CLC (black), CT-1 (grey), OSM (grey), LIF (grey), IL-27 (light grey) and IL-31 (lightest grey). CLC may require an additional α-receptor. The dominant STAT factor activations are marked in bold, however, this general assignment might not be true for all cells.
