# Supplementary material for: Interleukin-6 Signaling Pathway and Its Role in Kidney Disease: An Update
Source: Front Immunol. 2017 Apr 21;8:405. doi: 10.3389/fimmu.2017.00405 (PMC5399081; doi:10.3389/fimmu.2017.00405)
Supplement: Supplementary file 2 [file Image_2.PDF]

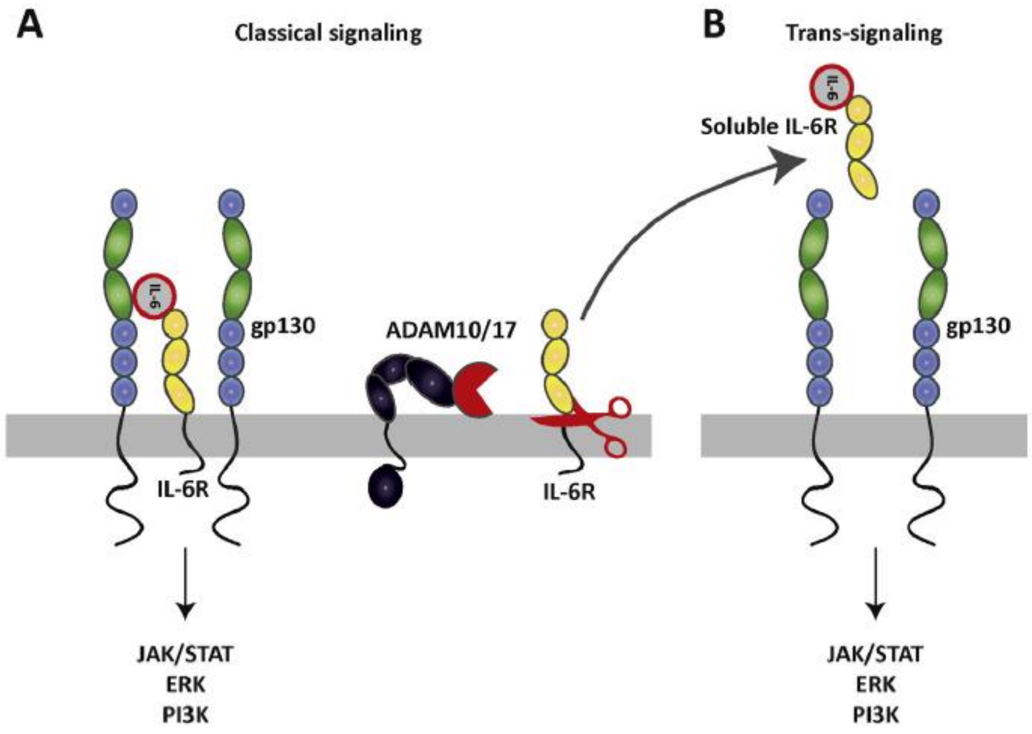

Supplementary figure 2. Overview of IL-6 classical and trans-signaling. (A) In classical signaling, binding of IL-6 to the membrane-bound α-receptor IL-6R causes the dimerization of its β-receptor gp130 which leads to down-stream JAK/STAT signaling in a restricted subset of cells. Shedding of the IL-6R by the ADAMs leads to the liberation of sIL-6R which can bind free IL-6. (B) This complex may then elicit JAK/STAT signaling in distal cells that express gp130 but lack the membrane-bound IL-6R in a process termed trans-signaling.
